# Positive Effects of a Short-Term Dog-Assisted Intervention for Soldiers With Post-traumatic Stress Disorder—A Pilot Study

**DOI:** 10.3389/fvets.2019.00170

**Published:** 2019-06-07

**Authors:** Andrea Beetz, Ira Schöfmann, Roland Girgensohn, Roger Braas, Christiane Ernst

**Affiliations:** ^1^Institute for Special Educational Development Support and Rehabilitation (ISER), Department for Special Education, University of Rostock, Rostock, Germany; ^2^Deptartment of Health Care (Distance Learning), IUBH University of Applied Sciences, Bad Honnef, Germany; ^3^Centre for Mental Health, Bundeswehr Central Hospital, Koblenz, Germany; ^4^Bundeswehr Medical Academy (SanAkBw), Munich, Germany; ^5^Bundeswehr School of Dog Handling (SDstHundeBw), Ulmen, Germany

**Keywords:** animal-assisted intervention, PTSD, military, animal assisted therapy, dog, trauma

## Abstract

Post-traumatic stress disorder (PTSD) is diagnosed in 3% of German and 14–16% of US military following deployment abroad. The treatment of PTSD in soldiers is often challenging and thus new, additional interventions supporting traditional trauma therapy are employed, like animal-assisted interventions (AAI). In this pilot study, 29 soldiers with PTSD received four sessions of 3 h once a week of dog-assisted intervention in addition to inpatient standard treatment at the military hospital, while the control group of 31 soldiers with PTSD received standard treatment only. The dog-assisted intervention sessions included a walk, different play and grooming activities and just relaxing together toward the end. What was new in our approach was that the AAI sessions were delivered by military personnel, military dog-handlers with their own dogs (either military or privately owned). Data on psychiatric symptoms, perceived stress, work and social life, and the therapeutic relationship were answered before the first AAI session, during the days following the last AAI session, 1 month later, and 3 months later. Only the intervention group also answered a questionnaire on trauma confrontation, consumption of alcohol/drugs, mental wellness, and perceived stress each week during intervention. Analyses showed a trend for worse values in work and social adjustment in the control group and a significant trend toward better values in the intervention group. On the other parameters differences between control and intervention group were not significant. The mental wellness of the intervention group improved over the 4 weeks of therapy, particularly regarding the ability to experience joy. There was no clear trend for perceived stress, but the relationship to the dog handler improved significantly over the course of the intervention. This is noteworthy in patients with PTSD who usually have difficulties trusting others, especially new people. Keeping in mind that the AAI took place only four times, our findings point toward the value of dog-assisted interventions. With a longer treatment period the positive effects and trends might become more distinct.

## Introduction

While many people will experience traumatic events during their lifetime, only some will develop a post-traumatic stress disorder (PTSD) or related problems, while others show resilience and can cope successfully. Certain populations are more likely to experience traumatic events, such as members of the military, firefighters, police, first aid, but also refugees or people living in war zones. Internationally, the military is faced with an increasing number of soldiers who receive the diagnosis of PTSD and is working on improving the therapeutic interventions for this group of patients. In the U.S., several organizations for veterans include animal assisted interventions (AAI), usually with horses or dogs, in their treatment programs, to alleviate symptoms of PTSD and to improve quality of life. Only few studies on the effects of AAI for PTSD in general, and for PTSD in veterans in particular, exist today. Therefore, a pilot project of the German military (Deutsche Bundeswehr) to support standard therapy for soldiers with PTSD via a short-term dog-assisted intervention was developed and evaluated; both will be reported here.

A brief introduction into the symptoms of PTSD and their treatment is given, followed by a short overview of effects of AAI in general and for PTSD. Also, possible underlying mechanisms explaining potential positive effects of human-animal interactions are addressed, prior to the report of the actual dog-assisted intervention and study.

## Post-traumatic Stress Disorder

### PTSD—Symptoms and Prevalence

In the DSM-5 (Diagnostic and Statistical Manual of Mental Disorders, 5th revision, APA) the acute stress disorder (ASD), also called acute stress reaction (ASR), and the post-traumatic stress disorder (PTSD) are both described as reactions toward a traumatic event. The acute stress disorder describes an acute stress reaction directly after a stressful, or even traumatic, experience and, in some cases, may also be present as a pre-stage of PTSD. For both diagnoses, the event needs to meet certain criteria to be considered traumatic. There is a wide variety of potentially traumatic experiences, including sexual or physical abuse, natural disasters, war, respectively large-scale acts of violence or accidents. For the diagnosis of PTSD, the experience must have included exposure to death or threat of death for oneself or others or the violation of one's physical integrity. The experience of the event must have been direct and accompanied by feelings of horror, fear and helplessness, and increased arousal and dissociation. In addition at least four of the following criteria need to apply: intrusive thoughts, flashbacks or nightmares, avoiding situations that could trigger the memory (and possibly re-experiencing), hyper arousal (those three are the main criteria for PTSD), and negative alterations in mood and/or cognition, which frequently relate to negative expectations about the future and the world in general, feelings of guilt, and self-blame.

Prevalence data for PTSD vary widely between countries and populations. While for US-American adults from the general population lifetime prevalence is estimated at 7–12% (1, 2), persons from regions with higher exposure to traumatic events (crisis regions, refugee camps, etc.) show occurrence rates of up to 58%, e.g., after combat exposure (2, 3). The prevalence rates for deployed US military personnel may be as high as 14–16% [for review see (4)].

In Germany, the lifetime prevalence for PTSD is estimated at 1.5–2.3%. However, some estimate it at 6% for men and 10% for women (5). For the German military prevalence rates of 2.9% within 1 year following the return from deployment abroad were documented (6). Wittchen et al. (6) also estimate that only 50% of German soldiers with PTSD receive a diagnosis and treatment.

### PTSD—Challenges in Treatment

There are several psychotherapeutic approaches in treating PTSD [for an overview see (7)], which frequently are accompanied by pharmacological treatment, mostly for comorbid disorders or specific symptoms as e.g., sleeping problems. In addition to general psychotherapeutic methods, such as building a trustworthy connection between patient and therapist, a phase of stabilization usually takes place before trauma confrontation. During the trauma confrontation, the patient is asked to mentally revisit the traumatic event (imaginal exposure) and reprocess it on several levels (cognition, emotion, physiological reactions). This is usually stressful for the patient and therefore requires a safe therapeutic environment.

The following challenges are frequently encountered in therapies with patients with PTSD: distrust toward other humans and the world in general, which is perceived as dangerous and threatening; life is seen as uncontrollable; the patient feels worthless and weak; loss of enjoyment. Many patients with PTSD therefore do not seek therapeutic help for a long time and this also poses a problem for family and friends. Social relationships are frequently broken off, leaving the patient without a supportive social network. Also, these symptoms make it more difficult to establish the necessary trustful therapeutic alliance, the basis of a successful therapy.

Evidence based psychotherapeutic approaches can effectively reduce symptoms of PTSD in veterans. For example, in a study by Monson et al. (8), 40% of veterans showed no PTSD symptoms anymore at the end of cognitive reprocessing therapy, and further 50% showed a significant reduction of symptoms. However, these improvements were only stable for a short period of time. In a controlled study with German veterans with PDTS, EMDR therapy proved effective in reducing symptoms (9). However, in their review, Steenkamp et al. (10) conclude that evidence-based therapy (cognitive-behavioral and trauma-focused) for PTSD is not as effective for veterans in comparison to other patient populations. One reason might be the difficult to establish therapeutic alliance and strong rejection of confronting the trauma. Steenkamp et al. (10) suggest that it is necessary to find alternative, additional approaches for the treatment of PTSD in soldiers.

## Animal Assisted Interventions

One form of intervention which recently has been combined with standard treatments of PTSD are animal-assisted interventions [AAI; for review see (11)], usually with horses or dogs, also in the treatment of soldiers with PTSD (12–14).

The International Association of Human Animal Interaction Organizations [(15), p. 5] defines AAI as a “goal oriented and structured intervention that intentionally includes or incorporates animals in health, education and human service (e.g., social work) for the purpose of therapeutic gains in humans. It involves people with knowledge of the people and animals involved.” It also distinguishes between Animal Assisted Therapy (AAT) and Animal Assisted Activities (AAA). While in AAT at least one person providing the AAI needs to be a trained therapist (either the animal handler or a person supervising the intervention), this is not required for AAA, which however still requires a certain level of training and expertise from the human-animal team.

### Human-Animal Interactions (HAI)—Effects Relevant for Trauma and PTSD

Research on the effects of interactions between humans and animals in general and in animal-assisted interventions provides a rationale for AAI supporting trauma therapy for soldiers with PTSD. The following effects of human-animal interaction (HAI), either assessed within the frame of AAI, experiments, or animal ownership, might be helpful in the treatment of patients with PTSD [for review and references not otherwise specified see (16)].

Well documented are the social effects of HAI. Humans of all ages, with and without clinical disorders, communicate more and more positively, verbally and non-verbally, in the presence of friendly animals. This is called the “social catalyst effect” of animals. Persons accompanied by a friendly looking animal receive more positive attention from others also, people in the company of animals (dogs) are trusted more by others. All these social effects of HAI could be important for therapy for soldiers with PTSD, because an animal involvement could facilitate establishing the necessary therapeutic alliance between therapist and client.

Among the psychological effects of HAI are an increase of the person's concentration and motivation in completing different tasks (17–19). Animal contact can reduce depression, anxiety, aggression and promote a positive mood and a sense of calmness, particularly before and during stress eliciting situations, including psychotherapeutic and medical treatments or exams. For patients with PTSD, who are usually anxious about confronting their trauma, these effects of HAI could be very helpful.

This reduction of psychological stress is also mirrored in the physiological effects: HAI has been shown to buffer human stress reactions, of the hypothalamic-pituitary-adrenal system (HPA-axis), as well as of the autonomic nervous system (ANS). Cortisol levels and cardiovascular parameters (heart rate and blood pressure) are positively influenced by HAI, with largest effects being found for stressful situations. The physical contact between the human and the animal seems to be especially effective, and also the level of familiarity with the animal, or the closeness of the relationship seems to be important, e.g., in increasing the level of the hormone oxytocin and the neurotransmitter dopamine.

### Processes Underlying the Positive Effects of AAI

The many different effects of HAI cannot be explained by one single theory. Their explanation requires a set of theories and processes, which are linked to each other [for review see (20)]. Some of these processes, which seem most important for the treatment of PTSD, will be briefly introduced here [for a more detailed report, see (20, 21)].

Julius et al. (22) describe the phenomenon that the mere presence of friendly, calm and resting animals can reduce psychological and physiological stress reactions and promote a feeling of security and a safe surrounding, which they call “biophilia effect.” This is probably due to subconscious perception of calm animals as a cue for a safe surrounding, based on biophilia (23). During the evolutionary history humans always lived together with animals in their surrounding and paying attention to them contributed to the survival of humans and promoted their fitness. This attention, or more general the affinity to animals and nature in general, is called biophilia (23).

Animals can contribute to human relaxation, not only via the biophilia effect, which requires no direct contact, but also via the activation of the oxytocin system, primarily via physical contact between human and animal. This seems to be a neurobiological mechanism which can explain many of the positive social, psychological and physiological effects of HAI (16, 22), since there is a large overlap between the effect spectrum of HAI and of oxytocin. The hormone and neurotransmitter oxytocin, which is also known as the attachment hormone, promotes social interaction and communication, mental and physiological relaxation, trust, a good mood, and reduces stress parameters (HPA axis and ANS) like blood pressure, heart rate and cortisol levels, and aggression. Its release is stimulated by e.g., massage, breastfeeding, but also other physiological stimulation like pleasant physical contact in general and touch (16, 24, 25). Research documented that persons petting a friendly dog show an increase in their oxytocin level and this effect is even larger if human and animal have a close relationship (26–28).

The activation of the oxytocin system seems to be a key for the explanation of additional positive effects of AAI supporting standard trauma therapy in a human-only setting. In AAI, nearly all patients want to pet the animals and therapy animals usually allow this kind of physical closeness and might even enjoy it. Even though this has not yet been investigated for patients with PTSD, it can be assumed from existing research documenting positive effects of AAI (11) that the activation of the oxytocin system might also occur in persons with this clinical disorder. While physical contact between therapist and patient is to be avoided in psychotherapy, and thus the oxytocin system cannot be activated this way, physical contact is an immanent part of AAI (29). Therefore, patients might develop trust toward their therapist (or the persons included in the AAI) easier and quicker, show a better mood, communication, relaxation, and motivation during sessions. These aspects are particularly important for trauma therapy, since patients are usually mistrusting, stressed and anxious. In addition, in AAI patients often show increased motivation, first to start a therapy at all, second, to attend the therapy sessions on a regular basis, and third, to more actively participate in the sessions. Wohlfarth et al. (18) describe the activation of intrinsic motivation via animal involvement, and propose that animals represent stimuli activating intrinsic motivation for most humans, probably also due to biophilia. Therefore, activities involving animals a person likes are carried out with more emotional involvement and energy.

Further processes which might be based at least partly on biophilia and the attention humans pay to animals, involve distraction (30), e.g., from the symptoms and negative thoughts, and mindfulness, meaning keeping one's attention in the here and now, not ruminating about the past or a negative future. Interactions with animals today are frequently found in psychotherapeutic treatments including mindfulness trainings, also for patients with PTSD who profit from such trainings since they might help to reduce intrusions and flashbacks. Traditional mindfulness trainings, however, are often difficult for patients with PTSD due to social anxiety in a group setting and the sleeping problems, which in turn cause concentration problems.

### Interventions Involving Dogs for Veterans With PTSD

During the recent years, several studies and reviews [e.g., (31)] document a high potential of interactions with dogs for veterans with PTSD. These interactions, however, have mostly been investigated empirically in veterans living with either companion dogs or a (psychiatric) service/assistance dog. Stern et al. (32) investigated 30 US military veterans with PTSD who lived with a dog. Self-reported positive effects, assessed retrospectively, included an increase in calmness and a decrease in loneliness, depression and worries about one's own and the family's safety. O'Haire and Rodruigez (33) report on the effects of service dogs as complementary treatment for PTSD in military personnel and veterans. In contrast to a group receiving usual care, the intervention group had improved in symptoms of PTSD after receiving a service dog, specifically higher quality of life, less depression and better social functioning. Rodruigez et al. (34) also found positive effects of living with a service dog on anger, anxiety, sleep disturbance and alcohol abuse in a population of veterans with PTSD, in contrast to veterans with PTSD on the waiting list for a service dog. Those veterans living with a service dog also displayed a higher cortisol awakening response, which the authors interpreted as a sign for better health and well-being in this population.

Owen et al. (35) describe canine-assisted therapy as a useful complementary or alternative medicine adjunct treatment for veterans with PTSD, based on a biopsychosocial rationale. Similarly, Yount et al. (36) argue that interactions with dogs may benefit veterans with PTSD in their treatment, based on neurobiological mechanisms, mainly the increase of oxytocin levels via contact with dogs.

However, no detailed description of such complementary adjunct dog-assisted interventions have been published, nor have they been evaluated. Therefore, our pilot project was aimed at both developing and implementing such a dog-assisted intervention and providing some empirical evidence. Another special feature of our project is the involvement of military service dogs and their handlers in delivering the dog-assisted intervention, in addition to the standard psychiatric treatment for veterans with PTSD.

## Research Question

Based on the knowledge about PTSD and its challenges in treatment and the potential positive effects of AAI supporting conventional trauma therapy/psychotherapy, the following research question was investigated: Does an AAI, delivered by military dog-handlers with their dogs supporting conventional standard treatment have additional positive effects for soldiers with PTSD in comparison to conventional treatment of PTSD without AAI?

## Method

To answer the research question, a controlled trial was conducted. The animal-assisted intervention which was evaluated had been developed for this purpose and had not been tested before. Therefore, the study has the character of a pilot study.

### Sample

The intervention group, which received the standard treatment plus the AAI (for a detailed description see below) included 29 patients (soldiers) of the Bundeswehr Central Hospital Koblenz, 26 men and three women, average age 38 ± 7 years (mean ± SD), with a diagnosed PTSD. Seven of those patients were single or divorced while 19 were married or lived with a partner. A higher level of school education was reached by eight patients, a medium level by twelve, and a lower degree by five participants (some patients did not give this information).

The control group consisted of 31 male soldiers with a diagnosed PTSD, who received the standard treatment, and were 38 ± 7 years of age. Seven of those patients were single or divorced while 23 were married or lived with a partner. A higher level of school education was reached by eight participants, a medium level by 15, and a lower degree by six participants.

Participants were asked for participation 2–3 weeks prior to the first AAI, when starting therapy at the hospital. Participation was voluntary. Written informed consent was obtained from all participants of this study.

Even though participants were not randomly selected for intervention or control group, they were comparable regarding age, level of education and relationship status. Data from the intervention group were collected during the spring and summer months, since the intervention with the dogs took place mostly outdoors. Each patient of the intervention group attended an AAI session of ca. 3 h, once a week, for a duration of 4 weeks, in addition to standard treatment. To avoid negative effects due to not receiving a special intervention while seeing that other patients did, data of the control group were collected the following year, during approximately the same time of the year. Patients were selected due to their voluntary participation, the diagnosis of a PTSD, absence of allergy to or fear of dogs, and being in psychotherapeutic treatment at the Bundeswehr Hospital Koblenz during the relevant time (stationary treatment for 6 weeks, during which the AAI took place, followed by ambulatory treatment; participants were in treatment for the entire data collection phase).

### Procedures

#### Dog-Assisted Intervention

Frequently in the field of AAI, the intervention is delivered by specially trained teams of a “therapy dog” and its handler. This handler is sometimes but not always a person with a therapeutic, educational or medical degree. However, such teams from outside the Bundeswehr were not approached due to several reasons. It was planned to continue the adjuvant dog-assisted intervention, if results of the pilot study would turn out positive, and thus to establish such teams within the Bundeswehr itself, because this has several advantages. Therefore, different from usual approaches in AAI, in our pilot-study soldiers handling a military service dog were selected to carry out the intervention. Being a soldier himself/herself, the dog-handlercan a more easily relate to the patient due to similar experiences, like deployment abroad and knowledge about the challenges of working in the military. This is especially important for soldiers with PTSD who mistrust most people. For this project it was also relevant that due to the geographical closeness of the Bundeswehr School of Dog Handling to the Bundeswehr Central Hospital Koblenz, a larger group of such service dog teams could be employed for AAI than external non-military teams were available in the direct surrounding.

For organizational reasons the dog-assisted intervention in our study was delivered to 10 patients at a time, who were driven from the hospital to the Bundeswehr School of Dog Handling together, once a week, for one morning (ca. 3 h of intervention time there; 4 h including transfer). Each patient worked with his/her service dog team on the premises of the School of Dog Handling, but apart from the other patients and their dog teams. While the 3 h are a much longer time of intervention than usually found in AAI, it should be mentioned that this was planned this way to give the patients enough time to adjust to the situation (being confronted again with uniforms, barracks etc. which had been avoided for a long time) and not feel rushed. Also different from other AAI, in our project the sessions did not involve therapy but rather aimed at improving general well-being, distracting from symptoms and providing the patients with experiences they had not had for a long time. Therefore, session included mainly a walk, play, grooming, feeding, and just relaxing together, and were partly standardized by these activities.

During the intervention units, patient and dog initially had the opportunity to get accustomed to each other and to familiarize themselves with the environment within the scope of a walk.

Subsequent “activation modules” were about performing a variety of exercises with the dog. This was done in the form of playful exercises from the field of dog sport, such as obedience, agility, dummy work, retrieval and search. In this context the service dog team was able to show its full individual repertoire (excluding rough tug-of-war games) and responding flexibly to a patient's wishes. The aim was to quickly develop a trusting relationship with the dog and—with some delay—also with the dog handler. By way of active movement, fun and joint activity and by talking about something harmless, an attempt was also made to find a distraction from the illness. Breaks between these modules were made when patient or dog needed them.

In a “slow-down module” at the end of the intervention unit, the physical contact with the “four-legged comrade” was explicitly promoted by having the patient perform tasks of maintenance and care on the dog (e.g., petting, brushing, bathing, feeding) to reduce stress parameters and contribute to mental relaxation.

The service dog handlers were instructed not to engage in conversations about the trauma or PTSD and change the subject when the patient would start to talk about these topics. The AAI was intended as a break from the trauma-focused therapy at the hospital. Furthermore, the dog handlers did not have the necessary therapeutic training. Also, the teams were instructed not to engage in any kind of fighting games between human and dog or work that involves aggression of the dog. Before the start of the intervention, the service dog handler teams received information about PTSD, what to avoid when dealing with patients with PTSD and what to do when symptoms like dissociation would occur.

Either a nurse or a therapist knowing the patients accompanied the patients to the Bundeswehr School for Dog Handling and stayed on site, but only got involved if a patient needed help (e.g., due to dissociation).

#### Selection of the Service Dog Teams

Since it is a new approach to employ the military service dogs and their handlers, soldiers, in this kind of dog-assisted intervention, the selection of the service dog teams for this project will be described in more detail. In contrast to common dog-handler teams in AAI, that have usually been tested by some larger organization in the field of AAI, most of the dogs in this study were trained for military purposes and thus needed to be tested for their suitability for the AAI.

All 18 service-dog teams that were involved in the project, volunteered for participation in the study. These were experienced service dog teams that had worked closely together usually for several years (at least for 7 months). The breeds of the dogs were Labrador Retriever or Malinois. Service dogs were selected for dog-assisted intervention against the background of minimizing the risks for patients, and maintaining the well-being of the animals. Due to their technical expertise and the detailed knowledge of their service dogs, the dog handlers are able to identify stressors and potential risks and to respond adequately. The selected dogs distinguished themselves by a high level of social competence and friendliness toward humans. Furthermore, in the run-up to deployment the selected teams had to undergo a character test and were, thus, certified for this special project. Dogs to be considered for this task needed to be social with humans in general, and attached to their handler, friendly, curious, trusting, calm, with strong nerves and stamina, even in surprising or scary situations. They needed to not mind being touched or cuddled, and to show a high tolerance for unusual human behavior (e.g., if flashbacks, dissociation should occur). It was important that the dog would not react aggressively even when feeling insecure or frustrated. Due to animal welfare concerns, strong avoidance behavior was an exclusion criterion for the dog-assisted intervention. There will always be a risk when working with dogs. To minimize this risk, the pre-selected dog was exposed to situations that are known to evoke aggressive behavior according to the “Training and Examination Regulations for Dog-Assisted Intervention of the Federal Armed Forces.” This is necessary for the safety of potential clients, the dog handler and the dog itself. In a continuing AAI project, the testing would be repeated at least once each year. The minimum age for a dog to take the test is 15 months. Passing the test is a requirement to be considered fit for service. Particularly, the test includes situations that the dog will be exposed to, to make sure that only especially suitable dogs will be chosen, regardless of the dog's breed. The “Training and Examination Regulations for Dog-Assisted Intervention of the Federal Armed Forces” include dogs' state of mental development, their conflict behavior and their willingness to work, especially with humans unknown to them. Testing takes place on one day and is divided into five tasks: (1) Everyday situations are created, e.g., being confronted with a person limping, stumbling, or walking using a white cane. A threatening situation is included, and the dog will be stared at. (2) For the second task, the dog has to demonstrate good obedience. It needs to return reliably when called, walk on a leash in an orderly fashion next to the dog handler, and wait for the dog handler in a specified place when told to. (3) Indoor situations: humans who cry, shout, make noise, embrace the dog without warning, or manipulate it physically. (4) For the fourth task, the dog is taken to inner city situations. The dog needs to be at ease with huge events and large numbers of people, as well as on public transport. (5) When confronted with other dogs, the dog needs to remain friendly or at least neutral. Overly aggressive behavior is not called for.

For each service dog team who had successfully completed the exam, an information sheet including a picture of the team was created informing about the respective characteristics of the service dogs and including some basic information about the handler. Thus, the therapists at the hospital could assign the service-dog teams in a targeted manner to the respective patients on basis of the described nature of the dogs and their knowledge about their patients.

### Instruments and Data Collection

The following questionnaires (base questionnaires) were given to the participants of the intervention and the control group. The intervention group answered these questionnaires once 2 days before the first AAI session (t1), during the days following the last AAI session (t2), 1 month later (t3), and 3 months later (t4). The control group answered the questionnaire set at corresponding times during their therapy.

- Questionnaire on sociodemographic data, military service (e.g., number and duration of deployment abroad), substance use (coffee, cigarettes, alcohol, other drugs) and current medication
- Hopkins Symptom Checklist 25 [HSCL-25; (37)]: This standardized questionnaire is a screening for psychiatric relevant symptoms, such as anxiety and depression over the course of the last 7 days. The 25 items are answered on a four-point Likert Scale; higher scores indicate more psychiatric symptoms.
- Perceived Stress Scale (PSS) 14 (38): This instrument assesses the subjective stress level, based on the premise that the intensity and duration of the subjective stress level is related to the likelihood of developing mental diseases. The 14 items are answered on a 5-point Likert scale, and higher scores indicate a higher stress load.
- Skala zur Einstufung des Arbeits- und Soziallebens [SEASL; Work and Social Adjustment Scale; (39)]: This instrument asks about functional problems with emotion and actions due to the PTSD. The 5 items can be endorsed on a scale reaching from 1 to 10, and higher scores indicate more problems.
- Fragen zur Therapeutischen Beziehung (Questions about the therapeutic relationship—self developed set of questions). The nine items are answered on a 5-point Likert scale; higher scores indicate a better therapeutic relationship. The internal consistency of this questionnaire is high, with Cronbach's alpha for positive affect between 0.854 and 0.986 at the different times and within the two groups.

Each questionnaire was evaluated by adding up the items. All patients who answered at least 15% of the questions of a questionnaire were included in its evaluation. Missing items were replaced with the average of the remaining items (this is standard practice in psychology and it is possible since all items of a given questionnaire are located on the same Likert scale).

Differences between intervention and control group at a given time were tested by independent *t*-test (after verifying normal distribution of the residues via Kolmogorov-Smirnov). Trends over the four measurements were to be tested by Page's trend test; however, it turned out that several participants of the intervention group did not turn in their final questionnaire (at t4). Since the Page test requires measurements at all time points, these participants would have to be excluded from the analysis. Therefore, it was decided to carry out the main analysis only for the first three time points with inclusion of all those participants who answered the corresponding first three questionnaires. The final time point t4 was then analyzed only as a secondary evaluation with all those participants who answered all four questionnaires.

Only the intervention group receiving the AAI answered the following additional weekly questionnaires to capture immediate, short-term effects of the dog-assisted intervention. These questionnaires were developed for this project, since no suitable instruments were available. Directly before leaving the hospital for the Bundeswehr School for Dog Handling where the AAI took place, the patients answered the “*weekly questionnaire T1*” about

- trauma confrontation during trauma therapy during the last 7 days, stressors besides the trauma therapy,
- consumption of coffee, cigarettes, alcohol and drugs during the last 7 days,
- questions about mental wellness during the last 7 days (eight items, higher values indicate more problems), and
- the Perceived Stress Scale 4 [PSS 4 (38); higher values indicate more stress].

In the morning following the day of the AAI the “weekly questionnaire T2” was answered, which asked about mental wellness during the last 2 days, any noticeable changes due to the dog-assisted intervention, and the relationship to the dog handler.

The mental wellness questionnaire had a Cronbach alpha of only 0.679 on the first day before the AAI; on the other dates the Cronbach alpha lay between 0.815 and 0.939. It was decided to accept the low Cronbach alpha for the first day and thus to leave the questionnaire unchanged (as removal of one or even two items would not have changed the Cronbach alpha for the first day significantly).

Again, each questionnaire was evaluated by adding up the items. All patients who answered at least 15% of the questions of a questionnaire were included in its evaluation. Missing items were replaced with the average of the remaining items.

Differences between before and after the intervention at a given time were tested by dependent *t*-test (after verifying normal distribution of the differences via Kolmogorov-Smirnov). Trends over the 4 weeks were tested by Page's trend test. In the case of these weekly questionnaires there was no problem with the fourth time point.

Always 2 days after the AAI, a group session under therapeutic supervision for all participants of the intervention group took place. After this session the questionnaire Stundenbogen für die Allgemeine und Differentielle Einzelpsychotherapie [STEP; (40), an instrument assessing the perceived quality of therapeutic sessions] was answered.

This research project was supported by the Bundeswehr Medical Service (Research-Project 13K1-S-901415). The project was reviewed with regard to patient welfare, data protection and animal welfare by the ethics committee of the State Chamber of Medicine of Rheinland-Pfalz, Germany [reference number 837.364.14 (9603)].

## Results

### Base Questionnaires

**Figure 1:**
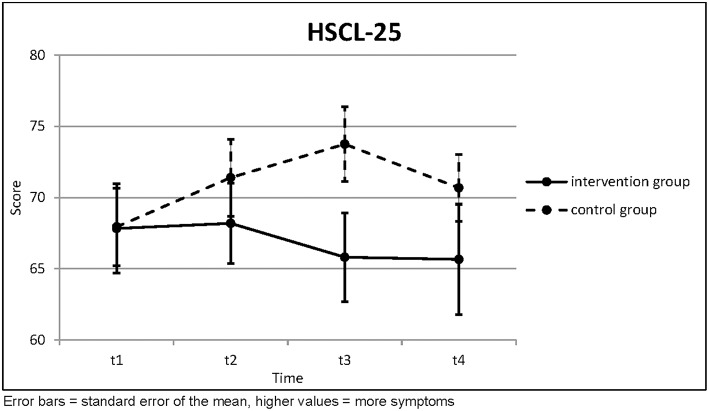
Hopkins Symptom Checklist 25.

Before the intervention, the symptoms, as measured by the Hopkins Symptom Checklist 25, were equal in both groups. During the course of the therapy, the intervention group improved slightly, whereas the control group deteriorated

(Table 1; Figure 1). The Page test was applied here with a null hypothesis of increasing symptoms and could not reject this with any statistical significance. The increase of symptoms in the control group was so pronounced, in fact, that had the Page test been applied with a *decreasing* null hypothesis, then it would have rejected this null hypothesis for the control group with very high statistical significance. The differences between the two groups were not statistically significant.

**Table 1:** Hopkins Symptom Checklist 25.

**HSCL-25**		**t1**	**t2**	**t3**	**Page's trend test (three points)**		**t4**	**Page's trend test (four points)**
Intervention Group (*n* = 16)	Mean	67.83	68.19	65.81	*L* = 191.0 *z* = −0.177	(*n* = 11)	65.66	*L* = 282.5 *z* = 0.783
	SD	12.575	11.238	12.438	*p* = 0.570		12.840	*p* = 0.217
Control Group (*n* = 31)	Mean	67.94	71.40	73.75	*L* = 346.5 *z* = −3.239	(*n* = 31)	70.68	*L* = 735.0 *z* = −2.489
	SD	15.156	15.049	14.543	*p* = 0.999		13.034	*p* = 0.994
Independent *t*-test	*t* =	0.025	0.750	1.858			1.102	
	*p* =	0.980	0.457	0.070			0.277	

*Higher values = more symptoms*.

**Figure 2:**
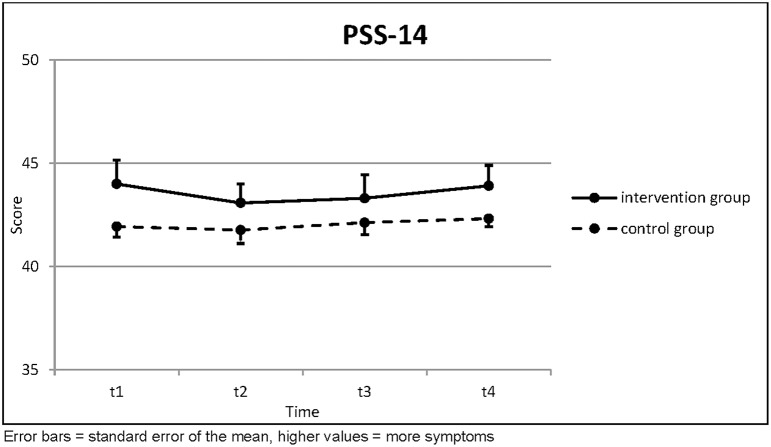
Perceived Stress Scale 14.

The perceived stress was generally higher in the intervention group than in the control group. It decreased a bit directly after the intervention in both groups and increased again in the following months (Table 2; Figure 2). None of this was statistically significant.

**Table 2:** Perceived Stress Scale 14.

**PSS-14**		**t1**	**t2**	**t3**	**Page's trend test (three points)**		**t4**	**Page's trend test (four points)**
Intervention Group (*n* = 13)	Mean	44.00	43.08	43.31	*L* = 156.5 *z* = 0.098	(*n* = 11)	43.91	*L* = 273.0 *z* = −0.209
	SD	4.183	3.353	4.131	*p* = 0.461		3.300	*p* = 0.583
Control Group (*n* = 31)	Mean	41.94	41.77	42.13	*L* = 372.0 *z* = 0.000	(*n* = 31)	42.32	*L* = 769.5 *z* = −0.342
	SD	2.816	3.640	3.274	*p* = 0.500		2.227	*p* = 0.634
Independent *t*-test	*t* =	1.913	1.107	1.008			1.781	
	*p* =	0.063	0.274	0.319			0.082	

*Higher values = more symptoms*.

**Figure 3:**
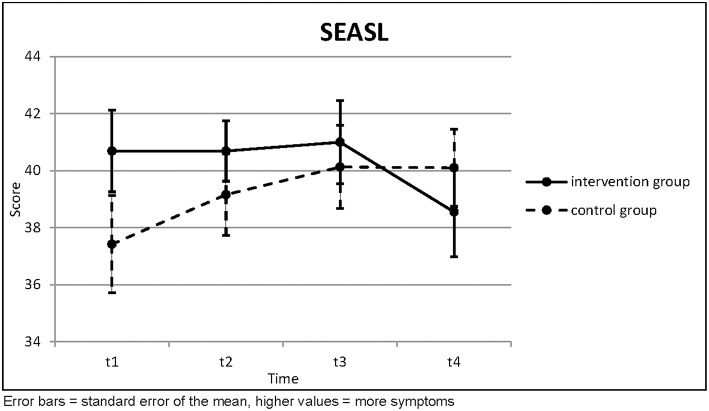
Work and Social Adjustment Scale.

The work and social adjustment had a trend toward worse values in the control group and a trend toward better values in the intervention group (Table 3; Figure 3). This last trend was statistically significant according to the Page test (*p* = 0.038) if all four questionnaires were included, because there was a sharp drop toward better values in the intervention group toward t4. Otherwise there was no statistical significance.

**Table 3:** Work and Social Adjustment Scale.

**SEASL**		**t1**	**t2**	**t3**	**Page's trend test (three points)**		**t4**	**Page's trend test (four points)**
Intervention Group (*n* = 13)	Mean	40.69	40.69	41.00	*L* = 154.0 *z* = −0.392	(*n* = 11)	38.55	*L* = 292.0 *z* = 1.776
	SD	5.170	3.816	5.276	*p* = 0.653		5.184	*p* = 0.038
Control Group (*n* = 31)	Mean	37.42	39.16	40.13	*L* = 364.5 *z* = −0.953	(*n* = 31)	40.10	*L* = 752.5 *z* = −1.400
	SD	9.472	8.000	8.119	*p* = 0.830		7.538	*p* = 0.919
Independent *t*-test	*t* =	1.170	0.656	0.355			0.629	
	*p* =	0.249	0.515	0.724			0.533	

*Higher values = more symptoms*.

**Figure 4:**
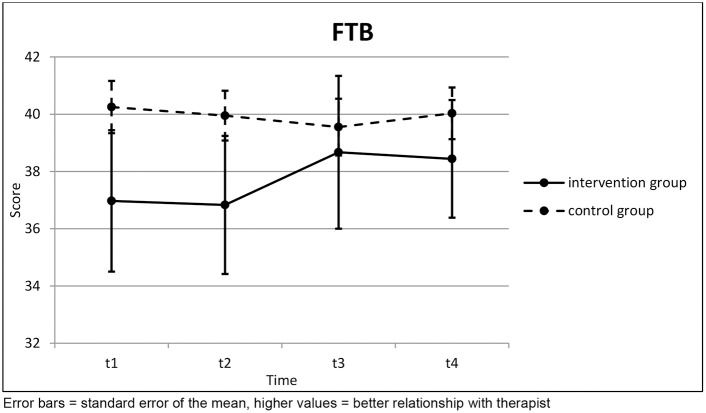
Questions about the Therapeutic Relationship.

There was no clear trend in the therapeutic relationship over the course of the therapy (Table 4; Figure 4). Accordingly, no statistical significance was found here.

**Table 4:** Questions about the Therapeutic Relationship.

**FTB**		**t1**	**t2**	**t3**	**Page's trend test (three points)**		**t4**	**Page's trend test (four points)**
Intervention Group (*n* = 12)	Mean	36.97	36.83	38.67	*L* = 151.0 *z* = 1.429	(*n* = 9)	38.44	*L* = 227.0 *z* = 0.231
	SD	8.555	8.354	9.247	*p* = 0.077		6.167	*p* = 0.409
Control Group (*n* = 25)	Mean	40.25	39.95	39.55	*L* = 293.0 *z* = −0.990	(*n* = 25)	40.03	*L* = 624.0 *z* = −0.069
	SD	4.568	4.347	4.952	*p* = 0.839		4.516	*p* = 0.528
Independent *t*-test	*t* =	1.530	1.500	0.378			0.819	
	*p* =	0.135	0.143	0.707			0.419	

*Higher values = better relationship with therapist*.

### Weekly Questionnaires (Intervention Group Only)

**Figure 5:**
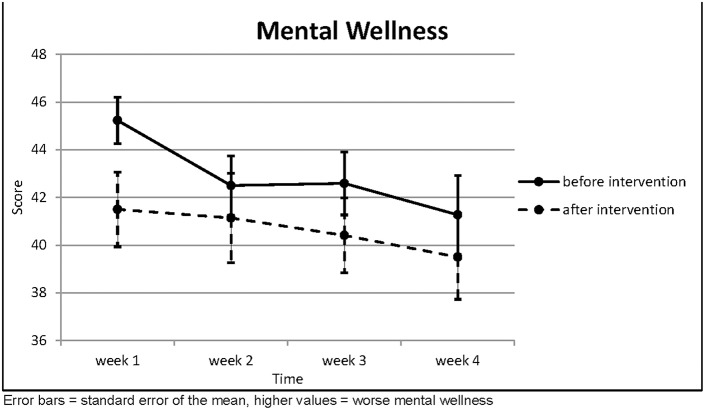
Questions about Mental Wellness.

The mental wellness improved over the 4 weeks of the intervention (Table 5; Figure 5); this trend was statistically significant for the values taken directly before the intervention (*p* = 0.007). The values taken on the morning after the intervention were always better than the values before (significantly so in the first and third week with *p* = 0.005 and *p* = 0.020, respectively), but they deteriorated again over time until the next therapy session. Among the items in this questionnaire was a question about the ability to experience joy. This item displayed the greatest improvement over the course of the therapy (*p* < 0.001).

**Table 5:** Questions about Mental Wellness.

**Mental wellness**	**(*n* = 22)**	**Week 1**	**Week 2**	**Week 3**	**Week 4**	**Page's trend test**
Before Intervention	Mean	45.23	42.50	42.59	41.27	*L* = 583.5 *z* = 2.474
	SD	0.969	1.244	1.322	1.657	*p* = 0.007
After Intervention	Mean	41.50	41.14	40.41	39.50	*L* = 562.0 *z* = 0.886
	SD	1.569	1.874	1.566	1.769	*p* = 0.188
Dependent *t*-test	*t* =	3.099	0.851	2.524	1.558	
	*p* =	0.005	0.404	0.020	0.134	

*Higher values = worse mental wellness*.

**Figure 6:**
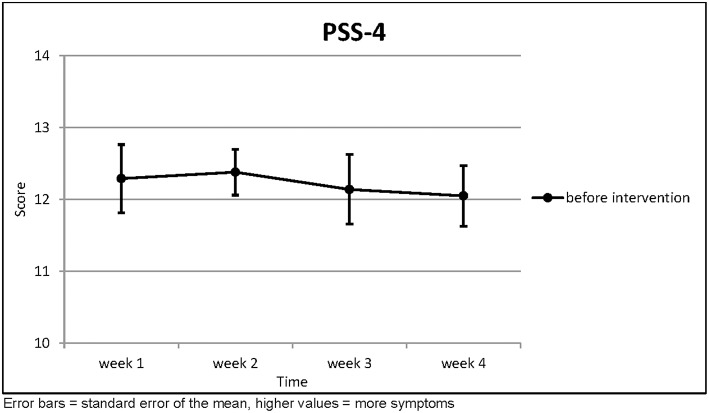
Perceived Stress Scale 4.

There was no clear trend for the perceived stress scores (Table 6; Figure 6). Accordingly, no statistical significance was found here.

**Table 6:** Perceived Stress Scale 4.

**PSS-4**	**(*n* = 21)**	**Week 1**	**Week 2**	**Week 3**	**Week 4**	**Page's trend test**
Before Intervention	Mean	12.29	12.38	12.14	12.05	*L* = 538.5 *z* = 1.021
	SD	2.171	1.465	2.220	1.936	*p* = 0.154

*Higher values = more symptoms*.

**Figure 7:**
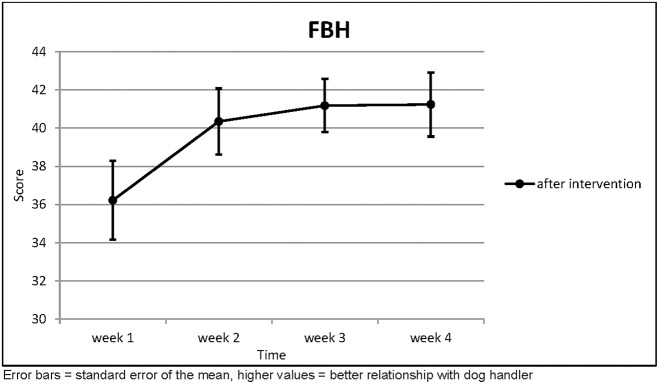
Relationship with the Dog Handler.

The relationship to the dog handler improved with statistical significance over the course of the intervention (*p* = 0.002; Table 7; Figure 7).

**Table 7:** Relationship with the Dog Handler.

**FBH**	**(*n* = 17)**	**Week 1**	**Week 2**	**Week 3**	**Week 4**	**Page's trend test**
After Intervention	Mean	36.22	40.35	41.18	41.24	*L* = 458.5 *z* = 2.815
	SD	7.432	6.224	5.028	6.051	*p* = 0.002

*Higher values = better relationship with dog handler*.

All of these results are reported without Bonferroni correction as this was a pilot study.

## Discussion

The comparison of intervention and control group over the four times of measurement did not indicate a significant advantage of the dog assisted intervention regarding PTSD symptom severity (HSCL-25), perceived stress (PSS-14), functional problems with emotions and actions due to the PTSD (SEASL), and the therapeutic alliance. Both groups did not show any significant improvement over time with regard to the investigated parameters. While this lack of a significant effect of the AAI in contrast to the control group is not what we expected, the lack of any improvement in neither group shows how difficult it is to achieve significant improvements for veterans with PTSD in the investigated time frame of 4 months. However, while there was no significant positive effect of the AAI or the standard treatment, neither on the full HSCL-25 nor on individual parameters, the control group in fact displayed a trend toward worse symptoms during and after the treatment. This was surprising, since other studies with veterans with PTSD from the German Bundeswehr document significant improvements of symptoms of PTSD and depression after 4 weeks of EMDR therapy (2–3 times per week) (9). Obviously, it would be important in future evaluations to assess in which phase of the treatment patients are. Since the AAI is aimed at improving motivation, trust, and by that the therapeutic alliance, the beginning of therapy would be the most suitable phase. Also, AAI over a longer period of time during the course of therapy, possibly with more frequent sessions, might produce further effects.

In our project, the dog assisted intervention only took place four times—a rather short intervention, leaving relatively little time for getting really acquainted and then already again having to prepare for an end of the meetings with the dog-team. However, the organizational framework (patients usually stay at the hospital for treatment for periods of 6 weeks, followed by ambulatory treatment) did not allow for more intervention units in this pilot project.

Thus, it is noteworthy that descriptive evaluation reveals less negative trends on the parameters under investigation for the intervention group than in the control group and stronger positive trends (improvement of symptoms etc.) in the intervention group. This suggests that there might be a positive effect of the dog-assisted intervention which possibly could reach statistical significance in further studies working with more AAI sessions over a longer intervention period (not per session but overall), and with larger samples of patients.

The weekly collected data from the intervention group also suggest some positive effects of the dog-assisted intervention. There were significant positive trends. Subjective well-being the day following the dog-assisted intervention was significantly improved in contrast to the morning before the AAI. Also, over the course of the 4 weeks well-being improved, and for the item “feeling happiness” in particular a large effect was found. Since the loss of enjoyment is a severe problem for patients with PTSD, this improvement, being able to feel “happy” again, at least once in a while, is an important effect of the AAI. Similarly important is the observation that also the relationship to the service dog handler improved significantly over the course of the 4 weeks. Since mistrust toward other persons is another problem of patients with PTSD, which makes establishment of a therapeutic alliance very difficult, this result points toward the opportunity such a dog-assisted intervention provides. Opening up the patient for trusting other beings again, maybe first trusting the dog, then the handler, could be utilized to support trusting the therapist as well and establishing the necessary therapeutic alliance for a successful trauma therapy. The free answers from the participants describing their experiences with the dog-assisted intervention further support our assumption that the contact with the animals can support positive mood, trust and other factors relevant for a good quality of life, even if this was not captured via the employed instruments and the controlled trial.

Obviously, this pilot project and study have limitations, first and foremost the low number of intervention units (four times) and the sample size. However, even with these limitations the positive findings point to a high likelihood that future, larger scale studies with a longer intervention period will find positive effects of the dog-assisted intervention in addition to standard treatment, also in comparison with a control group.

Overall, our data point toward a positive potential of dog assisted interventions supporting standard treatment for veterans with PTSD. Since treatment of this patient group is challenging, AAI may be an ideal candidate for an effective form of adjuvant interventions supporting trauma therapy.

Our result are further supported by the statements of the participants of this study about their experiences with the dog assisted intervention [these data have been reported in German in Ernst et al. (12); English translation by the authors]: e.g.,

- Via the interaction with the dog I was able to relax and calm down. He helped me to trust myself again, to be able to do at least some things right.
- I felt completely relaxed, as if I had no problems at all. I was able to laugh again and just be myself. The feeling of happiness was overwhelming, I have not felt that for a very long time. Really great!
- From my first contact with the dog until I had to say goodbye, I have not thought once about my PTSD and my problems.

## Data Availability

The datasets generated for this study are available on request to the corresponding author.

## Ethics Statement

The project was reviewed with regard to patient welfare, data protection and animal welfare by the ethics committee of the State Chamber of Medicine of Rheinland-Pfalz, Germany.

## Author Contributions

AB, IS, RB, and CE planned the project. CE, IS, and RB were responsible for conducting the AAI. AB and IS planned and conducted the evaluation. RG was responsible for the data analysis. Background and Method were written by AB, IS, and CE, the result section by RG, and the discussion by RG and AB.

### Conflict of Interest Statement

The authors declare that the research was conducted in the absence of any commercial or financial relationships that could be construed as a potential conflict of interest.
